# Editorial for the Special Issue: *Clostridium difficile*

**DOI:** 10.3390/microorganisms9020368

**Published:** 2021-02-12

**Authors:** Łukasz M. Grześkowiak

**Affiliations:** Institute of Animal Nutrition, Freie Universität Berlin, Königin-Luise-Str. 49, 14195 Berlin, Germany; lukasz.grzeskowiak@fu-berlin.de

*Clostridium difficile* (reclassified as *Clostridioides difficile* [1]), first described 85 years ago as a commensal microorganism in the feces of a newborn infant, has drawn further attention due to its involvement in intestinal infection that lead to high morbidity and mortality among individuals of different ages in healthcare and the community. It has also emerged as a zoonotic pathogen since highly genetically similar *C. difficile* isolates were characterized in humans, pets and farm animals [2,3]. Indeed, livestock can serve as a potential reservoir for *C. difficile* and pose a threat to humans since the spores facilitate the rapid spread of this bacterium in the environment. Although antimicrobial resistance is common among *C. difficile* [4], widespread antibiotic use in healthcare and animal husbandry has led to the development of multiresistant types, making the treatment of *C. difficile* infections (CDI) even more difficult [5]. Moreover, due to its anaerobic metabolism and relatively slow growth compared with other bacterial pathogens, research on *C. difficile* faces many challenges. However, in recent decades, significant advances in molecular methods has made possible a better understanding of the physiology and epidemiology of *C. difficile* in animals and humans [4,6,7].

*C. difficile* is one of the pioneer microbial colonizers in animals and humans. In farm animals such as pigs, an immature yet developing microbiota allows for the outgrowth of *C. difficile,* including toxigenic types, which may lead to infection [8,9]. It is noteworthy that concentrations of *C. difficile* and its toxins in neonatal piglets peak quickly during the first week and diminish from the second week onwards [9]. Therefore, a very short time window for *C. difficile* colonization, including toxin-producing types, can be observed. Such an observation is also true for infants. However, the lack of toxin-receptor expression or toxin-neutralizing antibodies at such early age has been suggested as a reason that infants are protected against CDI development [10]. On the other hand, in adult humans *C. difficile* outgrowth and infections have been associated with microbial dysbiosis in the gut caused by comorbidities, the use of antimicrobials or advanced age [11,12]. Recently, it has been suggested that a decreased expression of the High Mobility Group Box 1 HMGB1 cytokine in peripheral blood is involved in the diminished capability of elderly patients with CDI to fight the pathogen [7]. Indeed, reports show that microbial diversity is decreased in both *C. difficile*-colonized and CDI patients, and certain bacteria have been identified as playing a key role in colonization resistance against *C. difficile* and CDI development [13,14]. 

Lately, an in vitro study has demonstrated that sow colostrum, which contains antibodies against *C. difficile* toxins, may protect intestinal porcine cell lines from toxin-induced effects [15,16]. Moreover, the administration of antibodies against *C. difficile* toxins can protect all experimentally challenged piglets from developing systemic infection and minimize intestinal lesions [17]. Therefore, sufficient antibody titers against toxins transferred from mother to offspring through colostrum may be crucial in passive protection against gut intoxication and infection development once *C. difficile* colonization has been established in the piglet gut. In humans, clinical studies have identified protective antibodies against *C. difficile* toxins in the blood of patients who have suffered from CDI [18]. Further, a successful development of an antibody vaccine that neutralizes *C. difficile* toxin B has been demonstrated [19]. Finally, a recent study has identified a new immunoreactive surface protein isolated from *C. difficile* that is recognized by human blood and umbilical cord blood sera; therefore, it could serve a future use in preventive vaccine construction for humans [20].

The abovementioned studies demonstrate significant progress in the research on *C. difficile*. However, there are still many open questions regarding it, such as how healthy animal and human carriers of *C. difficile* and its toxins can remain free of symptoms or why there is a very short time window for colonization of *C. difficile* in certain farm animal species. Successful strategies to prevent and treat CDI in animals and humans while reducing the antimicrobial use are also needed. 
